# Plant growth-promoting traits of biocontrol potential bacteria isolated from rice rhizosphere

**DOI:** 10.1186/2193-1801-1-71

**Published:** 2012-12-18

**Authors:** Subramaniam Gopalakrishnan, HD Upadhyaya, Srinivas Vadlamudi, Pagidi Humayun, Meesala Sree Vidya, Gottumukkala Alekhya, Amit Singh, Rajendran Vijayabharathi, Ratna Kumari Bhimineni, Murali Seema, Abhishek Rathore, Om Rupela

**Affiliations:** 1 International Crops Research Institute for the Semi-Arid Tropics (ICRISAT), Patancheru, 502 324 Andhra Pradesh, India; 2 Department of Plant Breeding, CCS Haryana Agricultural University, Hisar, 125 004 Haryana, India

**Keywords:** Biocontrol, Plant growth promotion (PGP), Rice, Field evaluation, Rhizosphere bacteria

## Abstract

Seven isolates of bacteria (SRI-156, SRI-158, SRI-178, SRI-211, SRI-229, SRI-305 and SRI-360) were earlier reported by us as having potential for biocontrol of charcoal rot of sorghum and plant growth promotion (PGP) of the plant. In the present study, the seven isolates were characterized for their physiological traits (tolerance to salinity, pH, temperature and resistance to antibiotics and fungicides) and further evaluated in the field for their PGP of rice. All the seven isolates were able to grow at pH values between 5 and 13, in NaCl concentrations of up to 8% (except SRI-156 and SRI-360), temperatures between 20 and 40°C and were resistant to ampicillin (>100 ppm; except SRI-158 and SRI-178) but sensitive (<10 ppm) to chloramphenicol, kanamycin, nalidixic acid, streptomycin (except SRI-156 and SRI-211) and tetracycline. They were tolerant to fungicides benlate and captan, except SRI-158 and SRI-178, bavistin and sensitive to thiram (except SRI-156 and SRI-211) at field application level. In the field, four of the seven isolates (SRI-158, SRI-211, SRI-229 and SRI-360) significantly enhanced the tiller numbers, stover and grain yields, total dry matter, root length, volume and dry weight over the un-inoculated control. In the rhizosphere soil at harvest, all the isolates significantly enhanced microbial biomass carbon (except SRI-156), microbial biomass nitrogen and dehydrogenase activity (up to 33%, 36% and 39%, respectively) and total N, available P and% organic carbon (up to 10%, 38% and 10%, respectively) compared to the control. This investigation further confirms that the SRI isolates have PGP properties.

## Introduction

Plant growth − promoting rhizobacteria (PGPR) are the soil bacteria that colonize the roots of plants and enhance plant growth. PGPR can directly or indirectly affect plant growth through various mechanisms. Direct stimulation may include fixation of atmospheric nitrogen (Soares et al. 2006), synthesis of various phytohormones and enzymes (Patten and Glick 2002; Penrose and Glick 2003; Cheng et al. 2007) and solubilization of minerals in plants (Basak and Biswas 2009; Panhwar et al. 2012), while indirect stimulation includes inhibiting phytopathogens (Hao et al. 2011). Actively growing microbes are greater in number in the rhizosphere as crop plants release root exudates that produce, in addition to simple and complex sugars and growth regulators, different classes of primary and secondary compounds including amino acids, organic acids, phenolic acids, flavonoids, fatty acids, enzymes, steroids, alkaloids and vitamins (Uren 2000). Researchers around the world attempted to isolate PGPR organisms from the rhizospheres of crop plants.

Seven isolates of bacteria (SRI-156, SRI-158, SRI-178, SRI-211, SRI-229, SRI-305 and SRI-360), isolated from the rhizospheres of a system of rice intensification (SRI) fields, were earlier reported by us as having potential for biocontrol of charcoal rot of sorghum, caused by *Macrophomina phaseolina* (Tassi) Goid. and plant growth promotion (PGP) of the plant (Gopalakrishnan et al. 2011a). Also, the selected bacterial strains produced siderophore, indole acetic acid (except SRI-305), hydrocyanic acid (except SRI-158 and SRI-305) and solubilized (except SRI-360) phosphorous (Gopalakrishnan et al. 2011a). In the SRI method of rice cultivation, certain changes are done in the agronomic practices which include the use of much younger seedlings than are normally transplanted, planting them singly and carefully in a square pattern with wide spacing in soil that is kept moist but not continuously flooded and with increased amendments of organic matter and active aeration of the soil during weed control operation preferably with mechanical weeder (Uphoff 2003). The aim of the present study was to characterize and evaluate the PGP potential of seven SRI strains of bacteria in rice, grown under field conditions using the SRI protocols. It was also aimed to evaluate the potential of these PGP strains enhancing tolerance to salinity, pH, high temperature and resistance to antibiotics and fungicides.

## Materials and methods

### SRI bacterial isolates

A total of seven bacteria isolated from rhizosphere of a SRI fields, SRI-156 (*Pseudomonas plecoglossicida*; NCBI Accession Number: JQ247008), SRI-158 (*Brevibacterium antiquum*; NCBI Accession Number: JQ247009), SRI-178 (*Bacillus altitudinis*; NCBI Accession Number: JQ247010), SRI-211 (*Enterobacter ludwigii*; NCBI Accession Number: JQ247011), SRI-229 *(E. ludwigii*; NCBI Accession Number: JQ247012), SRI-305 (*Acinetobacter tandoii*; NCBI Accession Number: JQ247013) and SRI-360 (*P. monteilii*; NCBI Accession number: JQ247014), reported earlier by us as potential for biocontrol and PGP traits in sorghum (Gopalakrishnan et al. 2011a), were further studied in this investigation.

### Evaluation of SRI isolates for their physiological traits

#### Salinity

SRI isolates were streaked on Luria Bertani (LB) agar with various concentrations of NaCl ranging from 0% to 10% at the interval of 2% and incubated at 28°C for 72 h.

#### pH

The seven SRI isolates were streaked on LB agar, adjusted to pH 5, 7, 9, 11 and 13 and incubated at 28°C for three days. For pH 3, LB broth was inoculated with the seven SRI strains and at the end of 72 h incubation the intensity of growth was measured at 600 nm in a spectrophotometer.

#### Temperature

The seven SRI isolates were streaked on LB agar and incubated at 20, 30 and 40°C for 72 h. For 50°C, the LB broth was inoculated with the seven SRI strains, and at the end of 72 h incubation, the intensity of growth was measured at 600 nm in a spectrophotometer.

#### Antibiotic resistance/susceptible pattern

A total of six antibiotics viz. ampicillin, chloramphenicol, kanamycin, nalidixic acid, streptomycin and tetracycline were studied for their resistance/susceptible pattern against the seven SRI isolates. The required quantities of antibiotics were dissolved in sterilized Milli Q water and mixed into Nutrient agar just before pouring into the Petri plates (when the temperature of the media was about 50°C). Upon solidification, the SRI isolates were streaked and incubated at 28°C for 72 h.

#### Fungicide tolerance

SRI isolates were evaluated for their tolerance to fungicides at field application level. A total of four fungicides viz. benlate (benomyl 50%; methyl [1-[(butylamino) carbonyl]-1H-benzimidazol-2-yl] carbamate), captan (captan 50%; *N*-trichloromethylthio-4-cyclohexene-1, 2-dicarboximide), bavistin (carbindozim 50%; methyl benzimidazol-2-ylcarbamate) and thiram (dimethylcarbamothioylsulfanyl *N*, *N*-dimethylcarbamodithioate) were evaluated at field application levels at 3000, 2500, 4000, 3000 and 3000 ppm concentrations, respectively. The required quantities of fungicides were dissolved in sterilized Milli Q water and mixed into LB agar just before pouring into the Petri plates. Upon solidification, the SRI isolates were streaked and incubated at 28°C for 72 h.

Responses of the seven SRI isolates to salinity, pH, temperature, antibiotics and fungicide tolerance were recorded as follows: - = no growth; + = slight growth; ++ = moderate growth and +++ = good growth.

### Evaluation of SRI isolates for PGP traits on rice under field conditions

#### Study site

The experiment was conducted in Kharif 2011 (wet season) at ICRISAT, Patancheru, Andhra Pradesh, India (17^o^53”N latitude, 78^o^27”E longitude and 545 m altitude) with a medium duration rice variety, Sampada (135 days).^.^ Soils at the experimental site are classified as sandy loam in texture (55% sand, 17% silt and 28% clay) with alkaline pH of 8.5 − 9.0. The mineral content of the top 15 cm layer were as follows: available nitrogen 292 kg ha^-1^, available phosphorus 26 kg ha^-1^, available potassium 527 kg ha^-1^ and organic carbon 0.76 − 1.27%.

#### Design and treatments

The experiment was laid out in a randomized complete block design (RCBD) with three replicates and 10 m × 7.5 m subplots. Rice was grown by the system of rice intensification (SRI) method proposed by the Central Rice Research Institute (http://crri.nic.in). The seven SRI isolates were grown on a LB broth at 28°C for three days and further evaluated for their PGP traits. The control contained no SRI isolates.

#### Protocol for field experiment

Eleven-day-old single seedlings were uprooted from the nursery, their roots dipped in the respective SRI isolates broth (containing 10^8^ CFU ml^-1^) for 45 min and transplanted on 4th August 2011 at a spacing of 25 × 25 cm. Rice plants were inoculated with the SRI strains (1000 ml; 10^8^ CFU ml^-1^) once in 15 days until the flowering stage along with the irrigation water. The recommended dose of NPK (120, 60 and 40 kg ha^-1^, respectively) was supplied through compost and organic manures mixed with farm yard manure and rice straw. Water management was done as recommended for the SRI method, i.e. the alternate wetting and drying method of irrigation. After panicle initiation, all the plots were kept flooded with a thin layer of water (1–2 cm), and all were drained at 15 days before harvest. The crop was harvested manually on 23rd November 2011 and observed for plant height, tiller number, primary and secondary panicle number, panicle length, test seed weight, stover and grain yields and total dry matter. Root samples were collected from 0 to 30 cm soil profile and analyzed for root length density (EPSON expression1640×, Japan), volume and dry weight (dried in an oven at 70°C for 48 h). Soil samples were collected from 0 to 15 cm soil profile at harvest. These were analyzed for soil chemical analysis (% organic carbon, available phosphorous and total nitrogen as per the protocols of Nelson and Sommers 1982; Olsen and Sommers 1982 and Novozamsky et al. 1983, respectively) and biological analysis (dehydrogenase activity, microbial biomass nitrogen and microbial biomass carbon as per Casida 1977, Brooks et al. 1985 and Anderson and Domsch 1989, respectively).

### Data analysis

For each trait, data were analyzed by using Analysis of Variance (ANOVA) technique, by SAS GLM (General Linear Model) procedure (SAS Inst. 2002–08, SAS V9.3) considering replication and isolates as fixed in RCBD. Depth wise ANOVA was performed for the traits root length, root volume and root dry weight. Isolate means were tested for significance and compared using fishers protected least significant difference (lsd).

## Results

All the SRI isolates grew up to 4% and none grew at 10% of NaCl conditions. But the concentrations of 6% and 8% were critical as the isolates showed discriminatory performances in three NaCl concentrations. At 6% NaCl, the isolates SRI-156, SRI-211, SRI-229 and SRI-305 exhibited good growth and at 8% NaCl, the SRI isolates SRI-156 and SRI-360 did not show any growth whereas the others showed poor growth (Table 1). SRI isolates grown under varying pH showed that none grew till pH 3 and all of them exhibited good growth from pH 7 to pH 13. A pH of 5 was discriminatory for the isolates and the isolates SRI-156, SRI-229, SRI-305 and SRI-360 showed good growth (Table 1). Temperatures of 20 and 30°C were found optimum for the growth of all the isolates while at 40°C all but one (SRI-158) exhibited good growth. At 50°C, a single isolate (SRI-178) showed a moderate growth while others did not grow (Table 1). In the antibiotics resistance/susceptible pattern studies, all the isolates were found resistant to ampicillin (>100 ppm; except SRI-158 and SRI-178) but sensitive (<10 ppm) to chloramphenicol, kanamycin, nalidixic acid, streptomycin (except SRI-156 and SRI-211) and tetracycline (Table 1). When the SRI isolates were evaluated for their fungicide tolerance at field application level, they were tolerant to fungicides benlate and captan (except SRI-158 and SRI-178), bavistin but sensitive to thiram (except SRI-156 and SRI-211; Table 1).

**Table 1:** **Effect of salinity, pH, temperature, antibiotics resistance pattern and fungicide tolerance on the growth of SRI isolates**

Traits	SRI-156	SRI-158	SRI-178	SRI-211	SRI-229	SRI-305	SRI-360
**Salinity**							
4	+++	+++	+++	+++	+++	+++	+++
6	+++	++	++	+++	+++	+++	++
8	−	+	+	+	+	+	−
10	−	−	−	−	−	−	−
**pH**							
3	−	−	−	−	−	−	−
5	+++	+	++	++	+++	+++	+++
7	+++	++	+++	+++	+++	+++	+++
9	+++	+++	+++	+++	+++	+++	+++
11	+++	+++	+++	+++	+++	+++	+++
13	+++	+++	+++	+++	+++	+++	+++
**Temperature (°C)**							
20	+++	+++	+++	+++	+++	+++	+++
30	+++	++	+++	+++	+++	+++	+++
40	+++	+	+++	+++	+++	+++	+++
50	−	−	++	−	−	−	−
**Antibiotics resistance pattern (ppm)**							
Ampicillin	100	10	1	100	100	100	100
Chloramphenicol	5	2.5	10	10	10	10	10
Kanamycin	1	1	0	1	10	0	10
Nalidixic acid	10	1	0	10	10	0	10
Streptomycin	40	0	0	20	10	0	5
Tetracyline	10	1	10	10	5	10	5
**Fungicide tolerance**							
Thiram @ 3000 ppm	++	−	−	++	−	−	−
Bavistin@ 2500 ppm	+++	+	+++	+++	+++	+++	+++
Benlate @ 4000 ppm	+++	−	−	+++	+++	+++	+++
Captan @ 3000 ppm	+++	−	−	+++	++	+	++

+++ = good growth; ++ = medium growth; + = poor growth; - = no growth.

When the SRI isolates were evaluated in the field conditions for their PGP potential against an untreated control, the plots treated with SRI-158, SRI-211, SRI-229 and SRI-360 gave significantly greater grain yield, total dry matter and stover yield (Table 2). Also, these isolates significantly improved the tiller numbers (Table 2), however, no significant improvements were seen on plant height, numbers of primary and secondary panicles, panicle length and test seed weight (data not shown). The plots treated with SRI isolates significantly enhanced the root development at 0–15 cm depth including root length (except SRI-158 and SRI-305), root volume (except SRI-178) and root dry weight. At 15–30 cm depth, all the isolates significantly enhanced the root length and root volume but not root dry weight, though all the treatments enhanced the root dry weight, compared to the control (Table 3). Among the SRI isolates, SRI-229 caused greater increases of the yield parameters (including grain yield, stover yield and total dry matter) and root development (including root length, volume and dry weight).

**Table 2:** **Effect of SRI isolates on the morphology and yield potential of rice cultivation**

Treatment	No. of tillers (m^-2^)	Stover yield (g m^-2^)	Grain yield (g m^-2^)	Total dry matter (g m^-2^)
SRI-156	360	558	540	1098
SRI-158	523	685	643	1328
SRI-178	571	635	583	1218
SRI-211	470	663	595	1258
SRI-229	538	684	673	1357
SRI-305	662	605	628	1233
SRI-360	453	683	650	1333
Control	451	604	582	1186
Mean	504	640	612	1251
SE±	31.6***	19.7**	17.5**	30.0***
LSD (5%)	91.8	59.8	53.1	90.9
CV%	14	5	5	4

SE = standard error; LSD = least significant difference; CV = coefficient of variance; * = statistically significant at 0.05; ** = statistically significant at 0.01; *** = statistically significant at 0.001.

**Table 3:** **Effect of SRI isolates on the root development of rice at harvesting stage of rice cultivation**

	Root length (mm^-2^)		Root volume (cm^3^ m^-2^)		Root dry weight (g m^-2^)	
Treatment	0 − 15 cm	15 − 30 cm	0 − 15 cm	15 − 30 cm	0 − 15 cm	15 − 30 cm
SRI-156	5312	996	901	152	63.9	8.2
SRI-158	4979	711	859	106	60.2	5.9
SRI-178	5230	817	839	107	59.9	6.6
SRI-211	6542	781	1183	189	82.9	8.6
SRI-229	6389	922	1120	117	89.2	6.4
SRI-305	5004	887	903	112	66.9	6.4
SRI-360	5036	800	1065	112	72.9	6.7
Control	5019	509	840	89	47.1	5.6
Mean	5413	783	953	118	68.9	6.6
SE±	164.9***	87.1*	27.7***	11.1**	4.7**	1.1 ^NS^
LSD (5%)	516.9	274.2	86.9	35.3	14.9	-
CV%	4.6	16.9	4.4	13.9	10.4	25.1

SE = standard error; LSD = least significant difference; CV = coefficient of variation; NS = not significant; * = statistically significant at 0.05; ** = statistically significant at 0.01; *** = statistically significant at 0.001.

The biological activities, microbial biomass carbon (except SRI-156), microbial biomass nitrogen and dehydrogenase activity, in the top 15 cm rhizosphere soils at harvest were also found to be significantly higher in the SRI isolates-inoculated treatments over the un-inoculated controls (up to 33%, 36% and 39%, respectively; Table 4). The total N, available P, and organic carbon% were significantly higher in the top 15 cm of rhizosphere soils of SRI isolates treated plants (up to 10%, 38% and 10%, respectively) at harvesting than those of the un-inoculated control (Table 5).

**Table 4:** **Effect of SRI isolates on soil biological activity at harvesting stage of rice cultivation**

Treatment	Microbial biomass carbon (μg g^-1^ soil)	Microbial biomass nitrogen (μg g^-1^**soil)**	Dehydrogenase activity (μg TPF g^-1^soil 24 h^-1^**)**
Treatment			
SRI-156	2625	62	115
SRI-158	2876	71	120
SRI-178	3448	67	152
SRI-211	3129	80	133
SRI-229	3050	68	142
SRI-305	3776	75	131
SRI-360	3774	74	154
Control	2845	58	111
Mean	3190	69	132
SE±	177.3**	2.2***	8.6*
LSD (5%)	567.2	7.0	26.1
CV%	10	5	11

SE = standard error; LSD = least significance difference; CV = coefficient of variance; * = statistically significant at 0.05; ** = statistically significant at 0.01; *** = statistically significant at 0.001.

**Table 5:** **Effect of SRI isolates on soil chemical activity at harvesting stage of rice cultivation**

Treatment	Total nitrogen (ppm)	Available phosphorous (ppm)	Organic carbon (%)
SRI-156	2015	116	1.52
SRI-158	1967	121	1.48
SRI-178	2134	117	1.57
SRI-211	1930	106	1.61
SRI-229	2027	102	1.61
SRI-305	2072	91	1.51
SRI-360	1961	88	1.56
Control	1926	87	1.47
Mean	2004	103	1.54
SE±	21.3***	5.8*	0.027*
LSD (5%)	71.2	19.3	0.090
CV%	2	8	4

SE = standard error; LSD = least significance difference; CV = coefficient of variance; * = statistically significant at 0.05; *** = statistically significant at 0.001.

## Discussion

The SRI isolates used in the present investigation were earlier reported by us as having potential for biocontrol of charcoal rot of sorghum, caused by *M. phaseolina* and PGP of the plant (Gopalakrishnan et al. 2011a). In the present study, all the seven SRI isolates were able to grow at pH levels between 5 and 13, in NaCl up to 6% and temperatures between 20 and 40°C. SRI-178, identified as *Bacillus altitudinis* in our previous study (Gopalakrishnan et al. 2011a), was found to grow moderately even at 50°C, probably because of its spore-forming nature. Hence, it can be concluded that these isolates may have the ability to survive in the harsh environments such as saline and acidic to alkaline pH soils. Also, they were highly sensitive (<10 ppm) to chloramphenicol, kanamycin, nalidixic acid, streptomycin (except SRI-156 and SRI-211) and tetracycline and resistant to ampicillin (>100 ppm; except SRI-158 and SRI-178); these antibiotics could be used as markers for their identification in field evaluation studies. Further, all the SRI isolates were tolerant to bavistin, except SRI-158 and SRI-178 which were found tolerant to benlate and captan at field application level indicating their compatibility with fungicides, and hence can be a useful component of integrated pest and disease management program.

Four out of the seven isolates used in this investigation (SRI-158, SRI-211, SRI-229 and SRI-360) were found to significantly enhance growth parameters (including stover and grain yields, total dry matter, root length, volume and dry weight) and biological and chemical parameters (including microbial biomass carbon, microbial biomass nitrogen, dehydrogenase activity, total N, available P and% organic carbon). The mechanism by which the SRI isolates enhanced the morphological observations could be their PGP attributes such as indole acetic acid (IAA), siderophore production and phosphate solubilization (Gopalakrishnan et al. 2011a). IAA − producing microorganisms are known to promote root elongation and plant growth (Patten and Glick 2002), while siderophore producers act by binding Fe^3+^ from the environment and making it available to the plant (Wang et al. 1993). Free-living phosphate-solubilizing microbes release phosphate ions from sparing soluble inorganic and organic P compounds in soils and thereby contribute to an increased soil phosphate pool available for the plants (Artursson et al. 2006). The interaction between soil microorganisms and roots and their possible impacts on plant growth have been studied by Birkhofer et al. (2008) and Uphoff et al. (2009). When the soils were made wet and then dry, as in the case of the SRI method of rice cultivation, the levels of available P in the soil solution increased between 185% and 1900% as a result of population dynamics of species of phosphate-solubilizing bacteria and fungi (Turner and Haygarth 2001). Gayathry (2002) found that the counts of bacteria, such as the diazotrophs, *Azospirillum*, *Azotobacter* and phosphobacteria and microbial enzyme activity such as dehydrogenase, urease acid phosphatase, alkaline phosphatase and nitrogenase were significantly higher in SRI rhizospheres than those of the same variety of rice plants grown conventionally. In the present investigation, such enhanced activities were found only in the SRI isolates-inoculated treatments.

Colonization of roots by SRI isolates at the right place and time is essential for enhanced PGP activity. Successful interactions depend on sufficient population density, rhizosphere competence, root colonizing ability and PGP ability of the bacteria (Lugtenberg and Dekkers 1999). Although roots were not inspected for colonization in this study, the data on morphological (including roots), biological and chemical studies strongly suggest that SRI isolates had multiplied and colonized the inoculated rice roots. Hence, it can be concluded that the seven SRI isolates used in this study were apparently well adapted to the rice rhizosphere environment and enhanced the plant growth.

The seven isolates of SRI used in this study were apparently well adopted not only in the sorghum rhizosphere environment (Gopalakrishnan et al. 2011a) but also in the rice rhizosphere where they promoted plant growth. Hence, these isolates could be used as PGP agents in addition to biocontrol agents for the control of charcoal rot. The broad range of PGP and antifungal activities of the seven SRI isolates demonstrates multiple mechanisms of actions including antibiosis, production of cell wall degrading enzymes and plant growth − promoting hormones indicating its broad spectrum activity. Broad spectrum PGP and biocontrol agents (and their secondary metabolites) offer potentially effective novel strategies for controlling multiple pathogens and insect pests. A few of the available broad spectrum agents, mostly belonging to *Pseudomonas* spp., have shown broad spectrum antifungal activity by virtue of volatile and diffusible antibiotics (Hass and Keel 2003; Viji et al. 2003). Bacterial strains from diverse habitats of groundnut with broad spectrum PGP and antifungal activity have been isolated, identified and tested as a seed treatment for the control of collar rot in groundnut with or without thiram (Kishore et al. 2005). Secondary metabolites of *P. aeruginosa* possess antifungal, PGP and biocontrol activities (Bano and Musarrat 2003). ICRISAT has identified actinomycetes (isolated from various herbal composts) and bacteria that inhibit *Fusarium oxysporum* f. sp. *ciceri*, *Rhizoctonia bataticola*, *M. phaseolina*, *Helicoverpa armigera* and *Spodoptera litura* (Gopalakrishnan et al. [Bibr CR11_50][Bibr CR12_50][Bibr CR13_50]). The seven broad spectrum potential SRI isolates therefore are likely to be the potential candidates for the discovery of novel secondary metabolites which may be of importance for both PGP and biocontrol applications.
